# The Relationship Between Metabolic Syndrome Awareness and Perception of Health in Exercise Participants: A Cross-Sectional Study

**DOI:** 10.3390/medicina61030501

**Published:** 2025-03-14

**Authors:** Gökçe Avcu, Taner Akbulut, Emsal Çağla Avcu, Gian Mario Migliaccio

**Affiliations:** 1 Faculty of Sports Sciences, Institute of Health Sciences, Fırat University, Elazig 23119, Turkey; gokceavcu@hotmail.com; 2 Faculty of Sports Sciences, Department of Coaching Education, Firat University, Elazig 23119, Turkey; takbulut@firat.edu.tr; 3 Faculty of Sport Sciences, Department of Coaching Education, Sivas Cumhuriyet University, Sivas 58140, Turkey; emsalcagla.avcu@hotmail.com; 4 Department of Human Sciences and Promotion of the Quality of Life, San Raffaele Rome Open University, 00166 Rome, Italy; 5 Maxima Performa, Athlete Physiology, Psychology and Nutrition Unit, 20126 Milan, Italy

**Keywords:** metabolic syndrome, health, physical activity, lifestyle

## Abstract

*Background and Objectives*: The level of knowledge and awareness individuals have about diseases, as well as their health perceptions, can influence healthy lifestyle behaviors. The aim of this study is to evaluate the level of knowledge and awareness about metabolic syndrome (MetS) and the level of health perception, as well as to investigate the relationship between MetS knowledge and awareness and health perceptions. *Materials and Methods*: This research study was carried out using a cross-sectional survey model. The study group consisted of a total of 446 participants, including 328 men and 118 women, with an average age of 27.10 ± 8.86 years. In this study, the Metabolic Syndrome Knowledge and Awareness Scale (MSKAS) was used to assess metabolic syndrome awareness, and the Perception of Health Scale (PHS) was used to assess the perception of health. *Results*: The MetS knowledge and awareness level and the health perception of individuals attending sports centers are at a moderate level, and a weak but significant positive relationship has been found between MetS knowledge and awareness and health perception. Moreover, MSKAS scores were higher in women (*p* < 0.05). Participants who were knowledgeable about chronic diseases had higher MSKAS and PHS scores (*p* < 0.05). Those with a family history of chronic disease had higher PHS scores (*p* < 0.05). Participants who tracked their daily caloric intake had higher PHS scores (*p* < 0.05). Additionally, those who monitored their daily step count had higher MSKAS and PHS scores (*p* < 0.05). *Conclusions*: These findings suggest that the levels of MetS knowledge and awareness, as well as health perception, may vary depending on various individual and behavioral factors among individuals attending fitness centers.

## 1. Introduction

Metabolic syndrome (MetS) refers to a cluster of interconnected metabolic disorders, including obesity, hyperlipidemia, hypertension, elevated fasting blood glucose, and insulin resistance [1]. The underlying mechanisms of MetS involve complex interactions and encompass a combination of genetic predisposition, environmental factors, and lifestyle elements. Poor nutrition, physical inactivity, and high body mass are among the key triggers of this process [2]. MetS adversely impacts individuals’ quality of life by increasing the risk of type 2 diabetes, cardiovascular diseases, and mortality [3]. Lifestyle modifications play a critical role in reducing these risks. Specifically, exercise and healthy eating emerge as effective interventions in the prevention and management of metabolic diseases [4,5,6,7].

In the prevention and management of MetS, healthy nutrition, regular physical activity, and weight control are recommended as primary interventions [8]. However, adopting a healthy and active lifestyle may largely depend on knowledge and motivation levels. Adequate knowledge regarding the prevention and management of MetS can aid individuals in adopting and maintaining healthy lifestyle habits [9].

Research has shown that personal perceptions have a decisive impact on healthy behaviors [10,11]. Health perception refers to the emotional and cognitive evaluations individuals have regarding their health status. The adoption of healthy lifestyle behaviors is closely related to individuals’ perceptions of their health [12]. Individuals with a high health perception are generally more likely to make healthier lifestyle choices [13,14], which play a significant role in the prevention and management of chronic diseases.

Individuals’ perceptions of health and their knowledge and awareness of diseases can directly affect their tendency to engage in exercise, which is one of the healthy lifestyle behaviors. In this context, the current study aims to examine the knowledge and awareness levels of individuals attending fitness centers regarding MetS, as well as their health perception levels, and to investigate the relationship between MetS knowledge and awareness and health perception. The main hypothesis of this study is that there will be a significant relationship between knowledge and awareness of MetS and health perception among individuals who regularly attend fitness centers.

## 2. Materials and Methods

This research study was carried out using a cross-sectional survey model. All participants were informed about the purpose of this study in advance and provided written informed consent. This study was conducted in accordance with the Helsinki Declaration of Ethical Principles and was approved by the Fırat University Ethics Committee for Social and Human Sciences Research (13 August 2024/16).

### 2.1. Participants

In order to determine the required sample size, the G*Power (3.1) program was used. According to the findings of Aydemir et al. [15], with a Type I error (alpha) of 0.05, power (1-beta) of 0.8, an effect size of 0.33, and a two-tailed alternative hypothesis (H1), the minimum sample size required to detect a significant difference using this test should be at least 286. To obtain stronger results, 446 participants were included in this study.

The study group consisted of a total of 446 participants, 328 males and 118 females, with a mean age of 27.10 ± 8.86 years and living in the city center. Individuals who volunteered to participate in this study, attended the gym at least three times a week regularly, and were over 18 years old were included. Those who refused to participate, did not complete the data collection tools, or did not engage in regular exercise were excluded from this study.

### 2.2. Data Collection Tools

In line with the purpose of this study, the researcher created a ‘Personal Information Form’. This form included questions such as ‘age’, ‘gender’, ‘Are you knowledgeable about chronic diseases?’, ‘Does your family have a chronic disease?’, ‘Do you think you pay enough attention to your health?’, ‘Do you track your daily caloric intake?’, and ‘Do you track your daily step count?’.

### 2.3. Metabolic Syndrome Knowledge and Awareness Scale (MSKAS)

The validity and reliability study of the MSKAS was conducted by Karaman and Akbulut. This scale consists of 14 items and 4 sub-dimensions. The internal consistency (Cronbach’s alpha) of the scale was found to be 0.918, and in the present study, the internal consistency value was calculated as 0.930. The MSKAS is a 5-point Likert scale, where participants rate each item from 1 to 5. The lowest possible score on the scale is 14, and the highest score is 70. Higher scores, both in the sub-dimensions and the total score, indicate a higher level of knowledge and awareness regarding metabolic syndrome. The items in the scale are grouped into the following sub-dimensions: questions 1–5 under ‘Definition’, questions 6–8 under ‘General Health’, questions 9–11 under ‘Awareness’, and questions 12–14 under ‘Prevention’ [16].

### 2.4. Perception of Health Scale (PHS)

The Perception of Health Scale (PHS) was developed by Diamond et al. [17]. Its validity and reliability study was conducted by Kadıoğlu and Yıldız, and it has been translated into Turkish. The PHS is a 5-point Likert scale consisting of 15 items and 4 sub-factors. Negative items are reverse-scored. The scale has been applied to both nursing students and their families. The Cronbach’s alpha coefficient was found to be 0.77 for nursing students and 0.70 for families, showing good reliability in both groups [18]. In the present study, the internal consistency value was calculated as 0.681.

### 2.5. Statistical Analysis

Statistical analyses were conducted using SPSS 22 software. First, skewness and kurtosis values were examined to test the normality of the distribution. The skewness and kurtosis values for both scales were found to be within the range of −2 < … < +2, indicating that the data are normally distributed [19]. An independent-samples *t*-test was used for comparisons between two groups, and Pearson correlation analysis was used to determine the relationships between the two scale scores. The significance level was set to *p* < 0.05 for all tests. Correlation coefficients are interpreted as follows based on the reference ranges: low-level relationship (r = 0.10 to 0.29), moderate-level relationship (r = 0.30 to 0.49), and high-level relationship (r = 0.50 to 1.00) [20].

## 3. Results

As shown in Table 1, a statistically significant difference was found in the mean scores of Definition, Awareness, Protection, and total MSKAS scores according to the gender variable (*p* < 0.05). It was observed that this difference favored women in all sub-dimensions. There were statistically significant differences in the scores for Definition, General Health, Awareness, Protection, and total MSKAS score according to the response to the question ‘Are you knowledgeable about chronic diseases?’ (*p* < 0.05). It was observed that this difference favored the ‘Yes’ response in all sub-dimensions. Moreover, a statistically significant difference was found in the scores for Awareness and Protection according to the response to the question ‘Does your family have a chronic disease?’ (*p* < 0.05). It was observed that these differences favored the ‘Yes’ response. Furthermore, a statistically significant difference was found in the scores for Definition, General Health, and total MSKAS according to the response to the question ‘Do you think you pay enough attention to your health?’ (*p* < 0.05). It was observed that these differences favored the ‘Yes’ response in the sub-dimensions of Definition, General Health, and total MSKAS. As also shown in Table 1, a statistically significant difference was found in the scores for Protection according to the response to the question ‘Do you track your daily calorie intake?’ (*p* < 0.05). It was observed that these differences favored the ‘Yes’ response in the scores for Protection. Except for Definition (*p* > 0.05), statistically significant differences were found in all other sub-dimensions and total scores according to the response to the question ‘Do you track your daily step count?’ (*p* < 0.05). It was observed that these differences favored the ‘Yes’ response in all sub-dimensions.

As shown in Table 2, a statistically significant difference was found in the mean scores for Control Center according to the gender variable (*p* < 0.05). It was observed that this difference favored men. There was a statistically significant difference in scores for Importance of Health and for total PHS scores according to the response to the question ‘Are you knowledgeable about chronic diseases?’ (*p* < 0.05). It was observed that this difference favored the ‘Yes’ response in all sub-dimensions. There was a statistically significant difference in the scores for Control Center, Precision, and total PHS scores according to the response to the question ‘Does your family have a chronic disease?’ (*p* < 0.05). It was observed that these differences favored the ‘Yes’ response. There was a statistically significant difference in the scores for Precision, Importance of Health, and total PHS according to the response to the question ‘Do you think you pay enough attention to your health?’ (*p* < 0.05). It was observed that these differences favored the ‘Yes’ response in the sub-dimensions of Precision, Importance of Health, and total PHS. There was a statistically significant difference in the scores for Precision and total PHS according to the response to the question ‘Do you track your daily calorie intake?’ (*p* < 0.05). It was observed that this difference favored the ‘Yes’ response in the scores for Precision and total PHS. As also shown in Table 2, except for the Control Center sub-dimension (*p* > 0.05), statistically significant differences were found in all other sub-dimensions and total scores according to the response to the question ‘Do you track your daily step count?’ (*p* < 0.05). It was observed that these differences favored the ‘Yes’ response in all sub-dimensions.

In Table 3, the correlation analysis results indicate that there is a significant relationship between the Control Center subscale of the PHS and all subscales of the MSKAS (*p* < 0.05). Upon careful examination of the table, it is determined that all these relationships are positive (respectively, *r*: 0.224; *r*: 0.167; *r*: 0.183; *r*: 0.177; *r*: 0.226). A significant relationship is also found between the Precision subscale of the PHS and all subscales of the MSKAS (*p* < 0.05), with a positive direction (respectively, *r*: 0.187; *r*: 0.146; *r*: 0.185; *r*: 0.147; *r*: 0.198). Additionally, a significant relationship is found between the total score of the PHS and all subscales of the MSKAS, as well as the total score of the MSKAS, with a positive direction (respectively, *r*: 0.234; *r*: 0.168; *r*: 0.186; *r*: 0.178; *r*: 0.232).

## 4. Discussion

This study aims to assess the knowledge and awareness levels of individuals attending fitness centers regarding MetS and their health perceptions, as well as to analyze the potential relationship between these two factors. Considering the maximum possible scores from the scales, it can be stated that the participants exhibit a moderate level of both MetS knowledge and awareness and health perception. The research findings demonstrate that various demographic and health-related factors have a determining effect on individuals’ MetS knowledge and awareness levels, as well as on their health perceptions. Especially variables such as gender, knowledge of chronic diseases, family history of chronic illness, attitudes toward health consciousness, and daily calorie and step tracking are found to significantly influence MetS knowledge and awareness, as well as health perceptions. Additionally, the findings of this study suggest that there may be a weak but significant positive correlation between MetS knowledge and awareness levels and health perception.

In a study parallel to our findings, Özberk et al. found a moderate, significant positive correlation between osteoporosis awareness and health perception in women after surgical menopause [21]. These findings suggest that individuals with a knowledge and awareness of diseases may tend to perceive their health status more positively. However, in another study conducted by İzgüden and Gökkaya, no significant relationship was found between the total scores of the obesity awareness scale and the total scores of the health perception scale [22]. This finding suggests that the relationship between disease awareness and health perception may not always be evident and that it may vary across different disease groups or individuals with distinct demographic characteristics. Martell-Claros et al., in their study on patients diagnosed with diabetes and/or MetS, reported that participants had low cardiovascular risk awareness, and 42.2% of them assessed their health status as good or excellent [23]. This situation suggests that individuals may feel healthy even with a low awareness of their health status and that the relationship between health perception and awareness is not always linear.

The research findings indicate that knowledge about diseases and a family history of chronic illnesses have a significant impact on individuals’ awareness and health perceptions. This suggests that individuals’ perceptions of awareness and health risks may vary along with the information they have about their genetic history. The impact of a family history of chronic diseases on individuals’ health behaviors and risk perceptions is supported by numerous studies [24,25]. Grauman et al. found that individuals with no family history of myocardial infarction tend to perceive their cardiovascular risk as lower compared to those with a family history [26]. Studies have shown that individuals with a family history of diabetes are more knowledgeable about diabetes risk factors and are more likely to engage in healthy behaviors [27]. Knowing their genetic predisposition to diseases can motivate individuals to make behavioral changes to reduce their risks [28]. A family history of chronic diseases and deaths resulting from these diseases can influence individuals’ lifestyle changes and perceptions of disease risks [29].

Aydemir et al. conducted a study among coach candidates and found that women had higher levels of MetS knowledge and awareness compared to men [15]. Similarly, Aqel et al. found that female pharmacy students had higher levels of knowledge about MetS compared to their male counterparts [30]. Additionally, other studies on health perception have shown that women have higher health perceptions than men [31,32]. Our research findings support the conclusion that women have higher MetS awareness and health perception compared to men. These findings suggest that women may show greater interest in health issues and exhibit a higher tendency to seek information.

According to our research findings, variables such as attitudes toward paying attention to health, daily calorie tracking, and step tracking have been shown to affect MetS knowledge and awareness and health perception. These findings suggest that healthy lifestyle behaviors are one of the key factors influencing MetS knowledge and awareness and health perception. In a study conducted by Akeren et al., it was found that individuals with chronic diseases had MetS awareness levels above the moderate level, and one of the factors that increased MetS knowledge and awareness was adherence to diet [33]. İzgüden and Gökkaya found in their study that exercise frequency led to significant differences in obesity awareness and health perception. Participants who exercised more frequently (3–4 days per week) had higher levels of both obesity awareness and health perception compared to those who exercised less (0–2 days per week) [22]. Participation in activities that promote a healthy lifestyle is positively associated with individuals’ health knowledge [34]. Çıtak Tunç et al. identified a moderate positive relationship between health perception and healthy lifestyle behaviors among international university students [35]. Olgun and Kutlu found a weak positive correlation between health perception and healthy lifestyle behaviors [36]. Additionally, the moderate positive correlation between health perception and health awareness determined by Yıldırım and Çiftçi provides significant evidence of how health perception influences individuals’ health-related decisions [37].

This study has several limitations. First, due to its cross-sectional design, it is not possible to establish a causal relationship. More comprehensive longitudinal studies are needed to determine the progression of the observed relationships over time or to establish cause-and-effect links. Additionally, this study was limited to individuals who attend gyms in the city center of Elazığ, Turkey. This restriction limits the generalizability of the findings to a broader population or individuals from different geographical regions. The unique socio-cultural structure of Elazığ, gym participation rates, and local health awareness levels may have influenced the study results. Therefore, future research involving a wider range of demographic groups would contribute to a more comprehensive evaluation of the findings.

## 5. Conclusions

The findings of this cross-sectional study indicate a weak positive relationship between individuals’ knowledge and awareness of MetS and their health perception. Additionally, various demographic and health-related factors influence individuals’ knowledge and awareness of MetS as well as their health perception. However, since these findings only reflect a snapshot in time, longitudinal studies are recommended to further investigate this relationship over time.

## Figures and Tables

**Table 1:** Comparison of total and sub-dimension scores of MSKAS.

Variable	Definition Mean ± SD	General Health Mean ± SD	Awareness Mean ± SD	Protection Mean ± SD	MSKAS Mean ± SD
Gender					
Male (*n*: 328)	16.02 ± 4.51	9.67 ± 2.49	9.99 ± 2.80	10.41 ± 3.29	46.11 ± 11.13
Female (*n*: 118)	17.67 ± 4.53	10.19 ± 2.59	10.86 ± 2.76	11.58 ± 2.76	50.32 ± 10.68
	t = −3.402	t = −1.912	t = −2.897	t = −3.472	t = −3.559
	*p* = 0.001 *	*p* = 0.057	*p* = 0.004 *	*p* = 0.001 *	*p* = 0.000 *
Are you knowledgeable about chronic diseases?					
Yes (*n*: 305)	17.01 ± 4.75	10.05 ± 2.62	10.62 ± 2.83	11.19 ± 3.10	48.89 ± 11.50
No (*n*: 141)	15.27 ± 3.90	9.28 ± 2.23	9.35 ± 2.59	9.70 ± 3.10	43.61 ± 9.45
	t = 3.784	t = 3.034	t = 4.521	t = 4.727	t = 4.755
	*p* = 0.000 *	*p* = 0.003 *	*p* = 0.000 *	*p* = 0.000 *	*p* = 0.000 *
Does your family have a chronic disease?					
Yes (*n*: 190)	16.75 ± 4.80	9.90 ± 2.70	10.55 ± 2.87	11.10 ± 3.23	48.31 ± 11.90
No (*n*: 256)	16.25 ± 4.38	9.74 ± 2.39	9.97 ± 2.76	10.44 ± 3.11	46.41 ± 10.52
	t = 1.148	t = 0.656	t = 2.160	t = 2.160	t = 1.780
	*p* = 0.252	*p* = 0.512	*p* = 0.031 *	*p* = 0.031 *	*p* = 0.076
Do you think you pay enough attention to your health?					
Yes (*n*: 293)	16.77 ± 4.78	10.02 ± 2.64	10.38 ± 2.92	10.87 ± 3.26	48.07 ± 11.73
No (*n*: 153)	15.86 ± 4.09	9.40 ± 2.24	9.90 ± 2.58	10.43 ± 3.00	45.60 ± 9.79
	t = 2.014	t = 2.477	t = 1.711	t = 1.408	t = 2.223
	*p* = 0.045 *	*p* = 0.014 *	*p* = 0.088	*p* = 0.160	*p* = 0.027 *
Do you track your daily caloric intake?					
Yes (*n*: 195)	16.81 ± 4.98	9.99 ± 2.66	10.41 ± 2.86	11.16 ± 3.01	48.38 ± 11.44
No (*n*: 251)	16.19 ± 4.21	9.67 ± 2.41	10.07 ± 2.78	10.38 ± 3.26	46.32 ± 10.87
	t = 1.431	t = 1.331	t = 1.228	t = 2.593	t = 1.938
	*p* = 0.153	*p* = 0.184	*p* = 0.220	*p* = 0.010 *	*p* = 0.053
Do you track your daily step count?					
Yes (*n*: 189)	16.72 ± 5.43	10.19 ± 2.81	10.65 ± 3.04	11.32 ± 3.22	48.90 ± 12.80
No (n: 257)	16.27 ± 3.81	9.53 ± 2.26	9.90 ± 2.60	10.28 ± 3.07	45.99 ± 9.61
	t = 1.033	t = 2.751	t = 2.793	t = 3.484	t = 2.743
	*p* = 0.302	*p* = 0.006 *	*p* = 0.005 *	*p* = 0.001 *	*p* = 0.006 *

* *p* ˂ 0.05; SD = standard deviation.

**Table 2:** Comparison of total and sub-dimension scores of PHS.

Variable	Control Center Mean ± SD	Precision Mean ± SD	Self-Awareness Mean ± SD	Importance of Health Mean ± SD	PHS Mean ± SD
Gender					
Male (*n*: 328)	13.82 ± 4.76	11.35 ± 3.50	8.82 ± 1.74	7.22 ± 2.58	41.24 ± 8.14
Female (*n*: 118)	12.77 ± 4.60	11.74 ± 3.47	8.73 ± 1.70	6.75 ± 2.52	40.01 ± 7.15
	t = 2.063	t = −1.029	t = 0.494	t = 1.754	t = 1.444
	*p* = 0.040 *	*p* = 0.304	*p* = 0.622	*p* = 0.080	*p* = 0.126
Are you knowledgeable about chronic diseases?					
Yes (*n*: 305)	14.00 ± 4.84	11.80 ± 3.27	8.87 ± 1.65	7.46 ± 2.40	42.14 ± 6.97
No (*n*: 141)	13.33 ± 4.68	11.30 ± 3.58	8.77 ± 1.77	6.93 ± 2.54	40.34 ± 8.25
	t = −1.388	t = −1.396	t = −0.616	t = −2.069	t = −2.247
	*p* = 0.171	*p* = 0.150	*p* = 0.538	*p* = 0.039 *	*p* = 0.025 *
Does your family have a chronic disease?					
Yes (*n*: 190)	14.17 ± 4.94	11.94 ± 3.48	8.62 ± 1.97	7.12 ± 2.51	41.87 ± 7.90
No (*n*: 256)	13.08 ± 4.53	11.10 ± 3.46	8.94 ± 1.51	7.08 ± 2.51	40.20 ± 7.85
	t = 2.429	t = 2.542	t = −1.935	t = 0.184	t = 2.211
	*p* = 0.016 *	*p* = 0.011 *	*p* = 0.054	*p* = 0.854	*p* = 0.028 *
Do you think you pay enough attention to your health?					
Yes (*n*: 293)	14.07 ± 4.45	12.74 ± 2.90	8.84 ± 1.69	7.54 ± 2.21	43.20 ± 6.13
No (*n*: 153)	13.27 ± 4.87	10.79 ± 3.59	8.78 ± 1.75	6.86 ± 2.62	39.72 ± 8.45
	t = −1.685	t = −5.805	t = −0.336	t = −2.746	t = −4.519
	*p* = 0.093	*p* = 0.000 *	*p* = 0.737	*p* = 0.006 *	*p* = 0.000 *
Do you track your daily caloric intake?					
Yes (*n*: 195)	13.63 ± 4.82	11.86 ± 3.40	8.85 ± 1.66	7.22 ± 2.43	41.57 ± 7.53
No (*n*: 251)	13.44 ± 4.64	10.94 ± 3.55	8.73 ± 1.81	6.93 ± 2.59	40.06 ± 8.30
	t = −0.425	t = −2.753	t = −0.713	t = −1.206	t = −2.009
	*p* = 0.671	*p* = 0.006 *	*p* = 0.476	*p* = 0.228	*p* = 0.048 *
Do you track your daily step count?					
Yes (*n*: 189)	13.87 ± 4.65	11.89 ± 3.55	8.81 ± 1.61	7.31 ± 2.40	41.90 ± 7.37
No (*n*: 257)	13.11 ± 4.83	10.86 ± 3.60	8.79 ± 1.88	6.80 ± 2.62	39.57 ± 8.41
	t = −1.677	t = −3.108	t = −0.118	t = −2.150	t = −3.099
	*p* = 0.094	*p* = 0.002 *	*p* = 0.906	*p* = 0.032 *	*p* = 0.002 *

* *p* ˂ 0.05; SD = standard deviation.

**Table 3:** Correlation analysis of scale subscales and total scores.

Subscales		1	2	3	4	5	6	7	8	9
Definition (1)	*r*	1								
	*p*									
General Health (2)	*r*	0.671 **	1							
	*p*	0.000								
Awareness (3)	*r*	0.650 **	0.578 **	1						
	*p*	0.000	0.000							
Protection (4)	*r*	0.622 **	0.520 **	0.677 **	1					
	*p*	0.000	0.000	0.000						
MSKAS (5)	*r*	0.904 **	0.796 **	0.843 **	0.829 **	1				
	*p*	0.000	0.000	0.000	0.000					
Control Center (6)	*r*	0.224 **	0.167 **	0.183 **	0.177 **	0.226 **	1			
	*p*	0.000	0.000	0.000	0.000	0.000				
Precision(7)	*r*	0.186 **	0.146 **	0.185 **	0.147 **	0.198 **	0.503 **	1		
	*p*	0.000	0.002	0.000	0.002	0.000	0.000			
Self-Awareness (8)	*r*	0.040	0.009	0.022	0.038	0.035	0.031	0.025	1	
	*p*	0.405	0.853	0.646	0.424	0.467	0.518	0.596		
Importance of health (9)	*r*	0.028	0.004	0.031	0.005	0.003	0.045	0.006	0.169 **	1
	*p*	0.556	0.937	0.517	0.908	0.950	0.341	0.900	0.000	
PHS (10)	*r*	0.234 **	0.168 **	0.186 **	0.178 **	0.232 **	0.830 **	0.740 **	0.244 **	0.384 **
	*p*	0.000	0.000	0.000	0.000	0.000	0.000	0.000	0.000	0.000

** Correlation is significant at the 0.01 level (2-tailed). *r*: Pearson correlation.

## Data Availability

The raw data supporting the conclusions of this article will be made available by the authors on request.
